# Phytoliths in Pottery Reveal the Use of Spice in European Prehistoric Cuisine

**DOI:** 10.1371/journal.pone.0070583

**Published:** 2013-08-21

**Authors:** Hayley Saul, Marco Madella, Anders Fischer, Aikaterini Glykou, Sönke Hartz, Oliver E. Craig

**Affiliations:** 1 BioArCh, University of York, York, United Kingdom; 2 Institució Catalana de Recerca i Estudis Avançats, Institución Milá i Fontanals, Spanish National Research Council, Barcelona, España; 3 Danish Agency for Culture, Copenhagen, Denmark; 4 Institute of Prehistoric and Protohistoric Archaeology, University of Kiel, Kiel, Germany; 5 Stiftung Schleswig-Holsteinische Landesmuseen, Schloβ Gottorf, Schleswig, Germany; University of Pennsylvania, United States of America

## Abstract

Here we present evidence of phytoliths preserved in carbonised food deposits on prehistoric pottery from the western Baltic dating from 6,100 cal BP to 5750 cal BP. Based on comparisons to over 120 European and Asian species, our observations are consistent with phytolith morphologies observed in modern garlic mustard seed (*Alliaria petiolata* (M. Bieb) Cavara & Grande). As this seed has a strong flavour, little nutritional value, and the phytoliths are found in pots along with terrestrial and marine animal residues, these findings are the first direct evidence for the spicing of food in European prehistoric cuisine. Our evidence suggests a much greater antiquity to the spicing of foods than is evident from the macrofossil record, and challenges the view that plants were exploited by hunter-gatherers and early agriculturalists solely for energy requirements, rather than taste.

## Introduction

**Figure 1:**
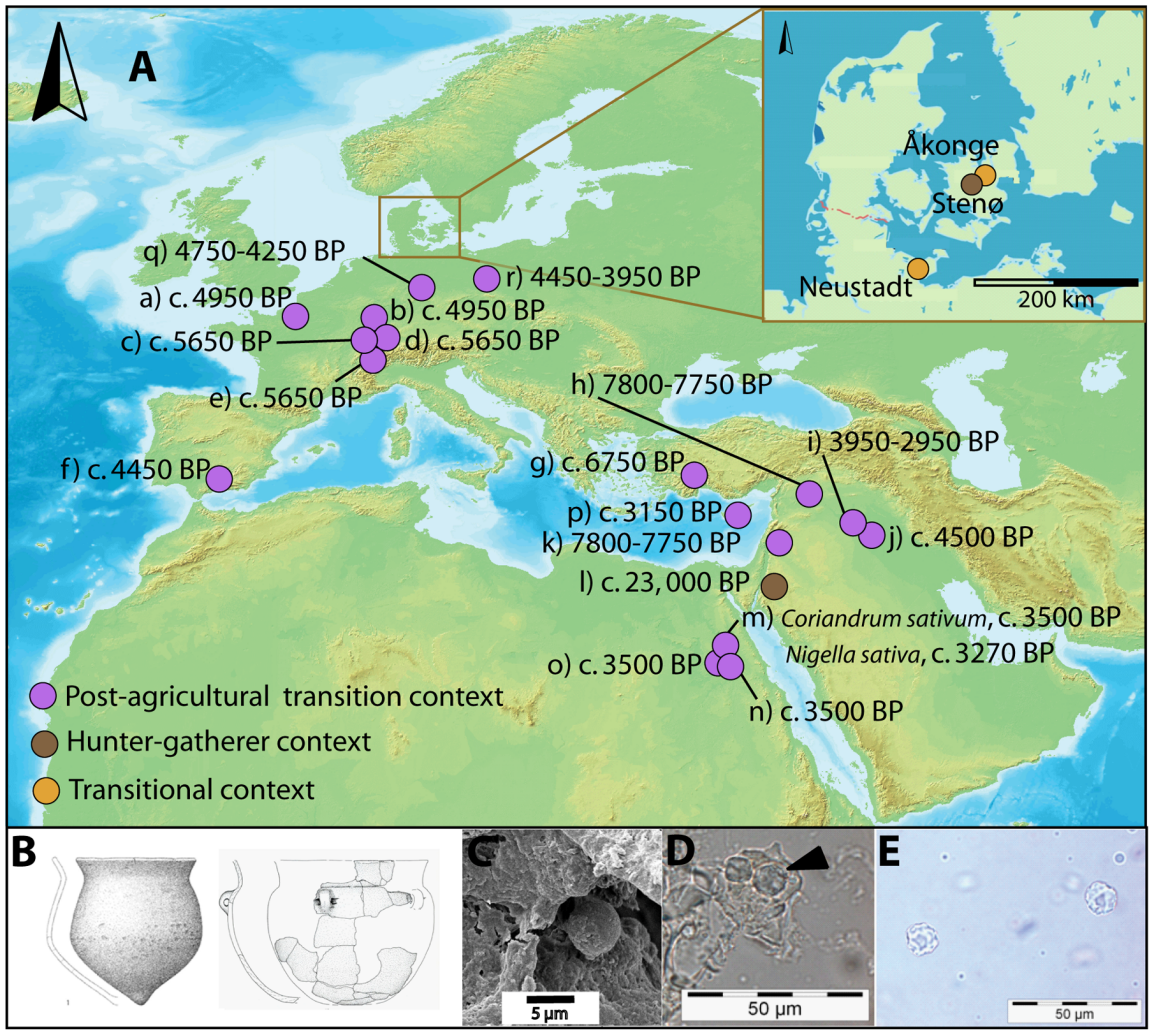
Early contexts from which spices have been recovered, with photomicrographs of globular sinuate phytoliths recovered from the pottery styles illustrated. Showing, A) A map of Europe showing an inset of the study area and sites from which the pot residues were acquired;, including also the Near East and northern Africa indicating early contexts where spices have been recovered: a) Menneville, France (*Papaver somniferum* L.), b) Eberdingen, Germany (*Papaver somniferum* L.), c) Seeberg, Switzerland (*Papaver somniferum* L.), d) Niederwil, Switzerland (*Papaver somniferum* L.), e) Swiss Lake Villages, Switzerland (*Anethum graveolens* L.), f) Cueva de los Murcielags, Spain (*Papaver somniferum* L.), g) Hacilar, Turkey (*Capparis spinosa* L.), h) Tell Abu Hureya, Syria (*Caparis spinosa* L.), i) Tell ed-Der, Syria (*Coriandrum sativum* L. and *Cuminum cyminum* L.), j) Khafaji, Iraq (Cruciferae family), k) Tell Aswad, Syria (*Capparis spinosa* L.), l) Nahal Hemar Cave, Israel (*Coriandrum sativum* L.), m) Tutankhamun's tomb, Egypt (*Coriandrum sativum* L.), n) Tomb of Kha, Egypt (*Cuminum cyminum* L.), o) Tomb of Amenophis II, Egypt (*Anethum graveolens* L.), p) Hala Sultan Tekke, Cyprus (*Capparis spinosa* L.), q) Heilbronn, Germany (*Papaver somniferum* L.), r) Zeslawice, Poland (*Papaver somniferum* L.) [compiled using 8–17]. B) Hunter-gatherer pointed-based vessel (on the left) and Early Neolithic flat-based vessel (on the right). C) Scanning Electron Microscope image of a globular sinuate phytolith embedded in a food residue, D) optical light microscope image of modern *Alliaria petiolata* globular sinuate phytoliths, and E) optical light microscope image of archaeological globular sinuate phytolith examples.

It has been plausibly argued that two of the most important events in world history were the nearly simultaneous voyages to America by Columbus and around Africa to India by Vasco da Gama [1]. Both these explorations were driven by a European desire for spice, documented in written sources from the classical period [2]. Such efforts culminated in the economic ethic of free-enterprise, colonialism and ultimately capitalism [3]. But is this *taste* for spice older? Classical texts testify to the widespread use of spices in European cuisine as far back in time as the 5th millennium BP [4]–[7]. In addition, archaeological studies of plant macrofossils have suggested that nutritionally poor but aromatically potent plants were available, and possibly used in cooking, in Neolithic Europe. The occasional preservation of seeds and peridermal tissues of plants, such as the opium poppy (*Papaver somniferum* L.), and aromatic herbs such as dill (*Anethum graveolens* L.), show that these spices spread from the Eastern Mediterranean, where their wild progenitors are found, to the Atlantic coastal margins c. 5,000 cal BP [8–17, Figure 1]. Earlier prehistoric evidence for the use of native European spices has been hard to demonstrate since seasonings originating from softer plant tissue can be invisible in charred fractions (e.g. leaves such as parsley *Petroselinum crispum* (Mill.) Fuss), or possible contenders are naturally abundant in the wild floral assemblages of excavated sediments. The current evidence is usually taken as support that Early Neolithic and pre-Neolithic uses of plants, and the reasons for their cultivation, were primarily driven by energy requirements rather than flavour [18].

The problem with identifying spices in the prehistoric record is twofold; first plant tissues only rarely preserve and second it is difficult to establish their culinary use. One line of research that is helping in understanding the origins of spice use is that of plant microfossil analysis. For example, starches are reported to survive well in carbonised and non-carbonised residues from a range of prehistoric tools and containers, as well as dental calculus [19]–[23]. In Asia, starch granules from spices, such as ginger and tumeric, have been extracted from nearly four and a half thousand year old Harappan cooking pots [24]. The recovery of phytoliths from carbonised deposits on the inside of potsherds offers the additional possibility to identify leafy, or woody seed material used as spices, which would not be detectable using starch analysis. Phytoliths charred by cooking have been found to be more resilient to destruction at pH extremes, ie. <pH3 and >pH9 [25]. Furthermore, the close association of phytoliths with cooking pots and with other organic traces of food within the charred deposit places the culinary interpretation beyond doubt. Here we report on the analysis of phytoliths from carbonised deposits adhering to the inside of Northern European cooking pots, dating from ca. 6,100 cal BP to 3,750 cal BP, and across the transition from hunting and gathering to farming.

Turning the resolution of analyses to the microscopic level opens a new prospect for documenting a wide variety of plant food. Phytoliths are rigid silica bodies produced by plants following the uptake of silicic acid (Si(OH)_4_) from the soil [25], [26], and a genetically and environmentally controlled deposition in the cells [27]. This deposition can happen in the cells of different plant tissues, often allowing for the identification of both the taxon and the utilised part [25], [28]. Phytolith research in prehistory has been successfully applied to understanding diet and past plant use in many parts of the world [e.g. 29–31]. Though rarely used as a culinary and paleoenvironmental indicator in northern Europe, phytoliths have made important contributions to debates about the role of plants in other temperate regions, such as China. Early origins (ca. 10,300 cal BP) for common millet (*Panicum miliaceum*) domesticates have been established on the basis of husk phytoliths in northern China [32], and rice cultivation has been suggested in 13,000 year old sedimentary sequences from the Yangtze River valley [33]–[35]. The applications of plant microfossil techniques, such as phytolith analysis, are also pushing back the date for the introduction of other important food products, like North American maize (*Zea mays*) [36]. So far, however, phytoliths have not been used to investigate the antiquity of non-staple crops.

## Results

Phytoliths were isolated from 26 out of 74 carbonised deposits recovered from the inside of pots from three sites in Denmark and Germany that span the transition to agriculture (see Materials And Methods) (Figure 1). Moreover, phytoliths were at significantly greater relative abundance from the interior carbonized deposits (n = 61) compared to exterior control deposits (n = 13), (*t = 1.99* p = <0.001), showing that they were the direct result of culinary practice (see Phytolith Counts, Figure S1). Globular sinuate phytoliths from seed epidermal tissues [37] (Figure 1) were found in eight archaeological samples, ranging in number from 1 to 32 per mg of deposit (Table 1).

**Table 1:** Phytoliths recovered from foodcrust on ceramic of EBK (Late Mesolithic Ertebølle culture) and TRB (Early Neolithic Funnel Beaker culture) style.

Site/sample	Vessel style	Globular sinuate phytoliths (counts mg^−1^)	Mean size (μm)	Max size (μm)	Min size (μm)
**Neustadt 2756_F**	EBK	31	7.2	10.5	4.3
**Neustadt 1495_F**	TRB	33	7.8	13.4	3.7
**Neustadt 3233_F**	TRB	30	10.3	15.4	6.8
**Neustadt 387_F**	TRB	32	8.1	12.0	5.5
**Neustadt 1193_F**	EBK	30	6.4	9.2	3.6
**Neustadt629_F**	EBK	30	8.5	16.3	5.0
**Åkonge 49.5/74.0_127_F**	EBK/TRB	1	8.9	8.9	8.9
**Stenø x004_201_F**	EBK/TRB	1	7.6	7.6	7.6
**Modern ** ***Alliaria petiolata***		50	6.9	11.2	4.8

Five samples are from the coastal site of Neustadt and include both pointed bottomed vessels, typical of the Late Mesolithic Ertebølle culture, and flat/rounded bottomed vessels typical of the Neolithic Funnel beaker culture (Figure 1). On the basis of lipid profiles and single compound isotopic characterisations these samples are associated with hunted resources such as marine, as well as terrestrial ruminant foods (Table 2). Taking a possible reservoir effect into consideration [38], their date is at least as old as c. 5,900 cal BP. This implies that they are probably contemporary with or younger than the local introduction of domesticated cattle and goat/sheep at 6,200–6,100 cal BP [39], but represent a use associated with hunted and gathered resources. Two further examples of ceramics associated with globular sinuate phytoliths were from pots found at the inland settlements of Åkonge and Stenø. The former derive from a context dating to c. 5,900 cal BP, which is contemporary with the earliest evidence of domesticated animals in this area [40]. The latter is from a context dated to ca. 6,100 cal BP, and thus is probably earlier than the regional introduction of domesticates and Neolithic pottery here. The residues on the surface of these wetland ceramics are less thick because the organic preservation has been compromised by modern drainage schemes, which may partly account for a reduced phytolith recovery compared to the samples from Neustadt.

**Table 2:** A summary of the major lipid classes identified in the samples where globular sinuate phytoliths occurred, including carbon stable isotope values of major fatty acids.

Site/Sample	Lipid classes detected	AMS dates	Fatty acid δ^13^C (‰)		Interpretation
			C_16:0_	C_18:0_	
**Neustadt 2756_F**	FFA, IFA, APFA MAG, cholesterol		−31.9	−36.0	Mixed ruminant/plant or ruminant/aquatic
**Neustadt 1495_F**	FFA (trace)	Associated charcoal, 5122+/−63 bp (6000– 5700 cal BP)	Not measured	Not measured	NA
**Neustadt 3233_F**	FFA, IFA, APFA MAG, DAG, cholesterol, dehydrobiatic acid		−19.7	−22.0	Mixed marine/plant resin
**Neustadt 387_F**	FFA, IFA, APFA		−25.6	−26.5	Marine
**Neustadt 1193_F**	FFA, IFA, APFA, cholesterol		−19.3	−19.8	Marine
**Neustadt 629_F**	FFA, IFA,APFA, cholesterol	Charred surface deposit, 5460+/−90bp (6450–6000 cal BP)Charred surface deposit, 5350+/−80bp (6300–5950 cal BP)	−22.9	−22.7	Marine
**Åkonge 49.5/ 74.0_127_F**	FFA, cholesterol		−29	−32	Ruminant
**Stenø x004_201_F**	FFA, cholesterol	*Cervus elaphus* bone from context, 5250+/−40 bp (6200–5950 cal BP)	Not measured	Not measured	Animal fat

FFA – Free fatty acids, IFA – Isoprenoid fatty acids, APFA – ω-(o-alkylphenyl)fatty acids, MAG – monoacylglycerols, DAG – diacylglycerols.

Based on the database of the BioPal collection (CaSES-Barcelona), augmented by 20 additional northern European specimens, totalling more than 120 European and Asian plants comprising stems, leaves, and seeds, morphologically equivalent phytoliths have been found only in the seed of modern garlic mustard (*Alliaria petiolata*,(M.Bieb) Cavara & Grande) (Figure 1) (average phytolith diameter 6.98 μm, range 4.8 μm to 11.2 μm). This plant is found from Europe to Central Asia, northern India and west China, and has a strong peppery, mustard flavour caused by the presence of volatile aglycones in both the edible leaves and seeds [41]. In addition to the phytoliths, all but one of the samples contained lipids from a range of marine and terrestrial animals [42, Table 2] as well as starchy plant foods [43]. These latter food types would provide the consumer with the bulk of the energy and macronutrient requirements, as garlic mustard has little nutritional value. At Neustadt, marine oils predominate in most samples with a lesser contribution from ruminant flesh, whilst ruminant and possibly other terrestrial animal products were spiced at the inland locations.

## Discussion and Conclusion

Despite the modest number of samples, it is demonstrated beyond doubt that the use of spice was practised regularly during the decades when domesticates were introduced in the western Baltic region. Although garlic mustard is a locally available source of spice, it is still uncertain if this practise was the result of Neolithic influence ultimately derived from the Near East, from where Old World farming originates, or if such advanced culinary practice was developed locally prior to the arrival of Neolithic elements in northern Europe. The ambiguity is partly due to problems in correction for reservoir effects in food residues where aquatic elements appear to be significant ingredients. In the western Baltic region a reservoir effect of up to 600 ^14^C years has to be accounted for in food derived from marine and freshwater systems [38], [44], [45]. The problem makes it challenging to determine if our samples, taken from the hunter-gatherer type pottery at Neustadt, are older than the earliest dates for domesticated animals and plants at the site.

There is no such problem with the context date for the sample from Stenø in Denmark. It clearly predates the introduction of domesticates to the area. Here however, there is only one radiocarbon date available, and it cannot presently be determined with certainty if this date is representative of the whole assemblage, including the ceramic sherd from which the sample was taken. Nevertheless, the present study demonstrates that plant microfossil analysis has opened a new avenue in the study of prehistoric culinary practice in northern European temperate climates. Further, it is now established that the habit of enhancing and altering the flavour of calorie rich staples was part of European cuisine as far back as the 7^th^ millennia cal BP.

## Materials and Methods

Permission was obtained from the museums of Holbæk, Kalunborg and Schleswig-Holstein for the removal of foodcrust samples from the sherds. These samples were donated to the project for destructive study. Foodcrusts were scraped from sherds using a clean scalpel. Weighed residues (∼1 mg) were treated with H_2_O_2_; 10%, 10 ml; 15–30 min and disaggregated. Samples were centrifuged (2665 RCF; 3 min) and the supernatant removed. The remaining residues were washed three times with UltraPure water and made up to 1ml suspensions. The supernatant, containing liberated phytoliths was added to microscope slides and left to dry (18°C). Samples were mounted in glycerol before viewing in rotated planes using an Olympus IX71 inverted microscope (*Olympus, UK*) fitted with a ColorView III camera *(Olympus, UK)* linked to Digital Image Solutions program CellD 2.6 (Build 1200) (*Olympus, UK*). Silica body counts were normalised and reported per mg of carbonised deposit. Interior (F) and exterior (S) silica body counts were compared using a two-tailed t-test, to establish whether there were significantly higher numbers on the interior, indicative of a deliberate packing of the pots with plant food. Identifications were not carried out on samples with <33 counts mg^−1^, which corresponds to the maximum count on the exterior deposits.

The lipid analysis followed established protocols [46]–[48]. A total lipid extract (TLE) was obtained through solvent extraction of either ceramic powder (approximately 1 g), drilled from the interior surface of each potsherd, or crushed surface residue (15 mg). An aliquot of each TLE was silylated and analysed by gas chromatography-mass spectrometry (GC-MS). Another aliquot of the TLE was methylated for the analysis of fatty acid methyl esters (FAMEs). An aliquot of the FAME fraction was analysed by GC-MS analysis and another aliquot by GC-combustion-isotope ratio MS (GC-c-IRMS) to obtain a δ^13^C value for the two major saturated free fatty acids, with 16 and 18 carbon chain lengths.

Radiocarbon dates from Neustadt were made on both charcoal associated with the vessel N_1495 (5122±63 bp, 6000–5700 cal BP (2σ) (KIA-39760)), and on charred foodcrust from N_629 (5460±90 bp, 6450–6000 cal BP (2σ) (AAR-11409), 5350±80 bp, 6300–5950 cal BP (2σ) (AAR-11410)). At Stenø, the context from which the pottery derived was dated to 5250±40 bp, 6200–5950 cal BP (2σ) (Poz-31049) using terrestrial mammal bone. Three directly dated samples of carbonised material from the ceramic matrix were made at Åkonge: ‘Peter’s Pot’ (5140±70 bp, 6200–5700 cal BP (2σ) (AAR-4395)) [40], 49.5/77.0:18 (5155±40 bp, 6000–5800 cal BP (2σ) (AAR-4817)), and 49.5/77.0:26 (5095±45 bp, 5950–5750 cal BP (2σ) (AAR-5363)). A further four radiocarbon dates were made on ‘sooty’ exterior deposits from Åkonge: 49.5/77.5:10 (5140±40 bp, 6000–5800 cal BP (2σ) (AAR-5111), 50.0/75.5:18 (5070±45 bp, 5950–5750 cal BP (2σ) (AAR-5113), 49.5/77.0:18 (5195±40 bp, 6200–5900 cal BP (2σ) (AAR-4816)), and 49.5/77.0:26 (5195±45 bp, 6200–5800 cal BP (2σ) (AAR-5109)) [38]. In addition several samples of terrestrial mammal bone, including bones of domestic cattle, were dated within the time interval 5120±40 to 4950±60 bp, 5980–5810 cal BP (2σ) [44].

## Supporting Information

Figure S1 There is a significant difference (*t = 1.99* p = <0.001) in phytolith counts between interior carbonised (n = 61) and exterior soot (n = 13) supporting the claim that vessels with high counts were from the deliberate preparation of plants within the ceramics. The graph shows those samples with high silica body counts (>33 mg^−^^1^, green columns) that qualified for further phytolith identification analysis.
(TIF)
