# Supplementary figures and images for: Phytoliths in Pottery Reveal the Use of Spice in European Prehistoric Cuisine
Source: PLoS One. 2013 Aug 21;8(8):e70583. doi: 10.1371/journal.pone.0070583 (PMC3749173; doi:10.1371/journal.pone.0070583)

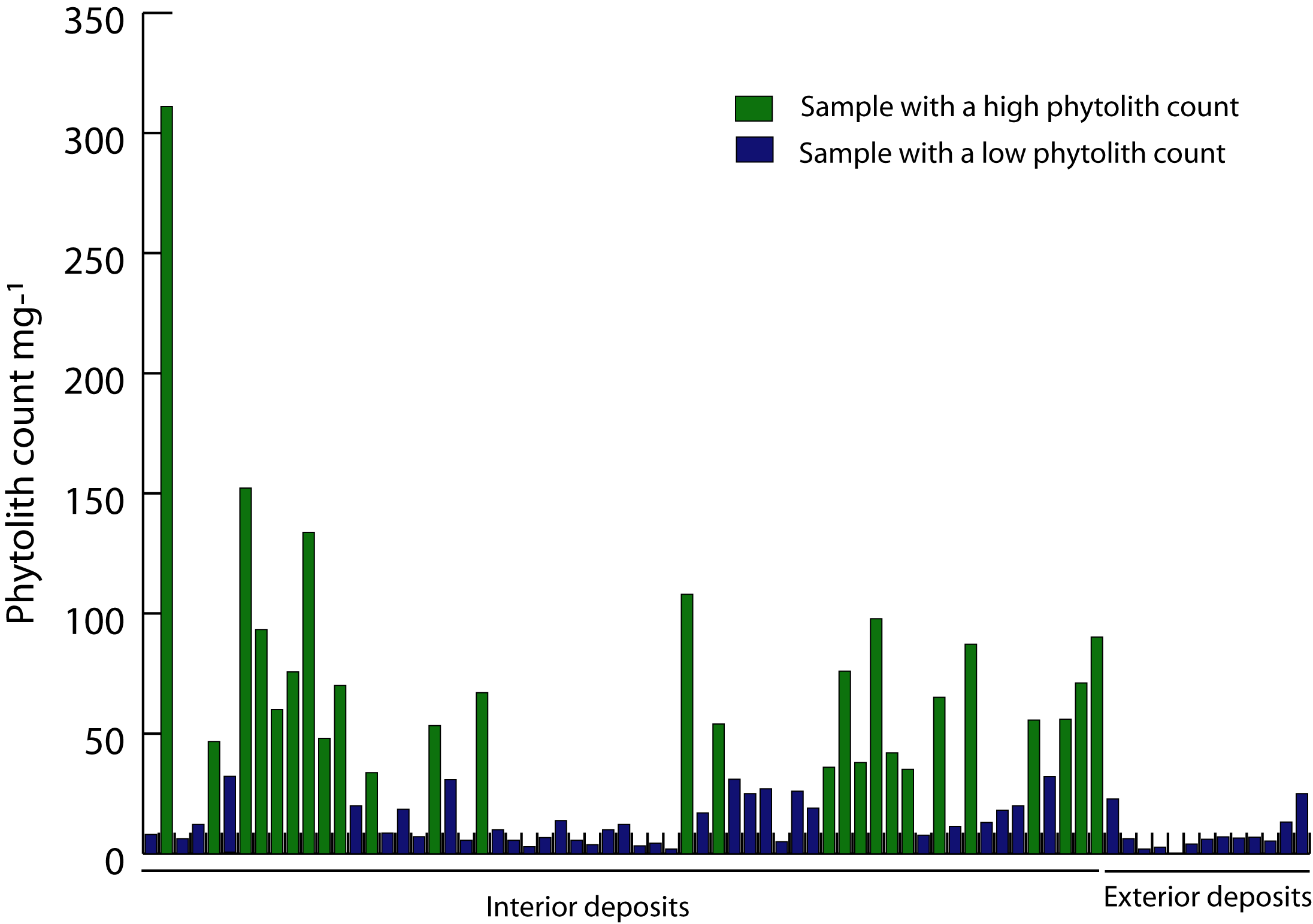

Supplement: Figure S1 — There is a significant difference (t = 1.99 p = <0.001) in phytolith counts between interior carbonised (n = 61) and exterior soot (n = 13) supporting the claim that vessels with high counts were from the deliberate preparation of plants within the ceramics. The graph shows those samples with high silica body counts (>33 mg− 1, green columns) that qualified for further phytolith identification analysis. (TIF) [file pone.0070583.s001.tif]
